# Up-Regulation of TLR7-Mediated IFN-α Production by Plasmacytoid Dendritic Cells in Patients With Systemic Lupus Erythematosus

**DOI:** 10.3389/fimmu.2018.01957

**Published:** 2018-08-28

**Authors:** Kei Sakata, Shingo Nakayamada, Yusuke Miyazaki, Satoshi Kubo, Akina Ishii, Kazuhisa Nakano, Yoshiya Tanaka

**Affiliations:** ^1^First Department of Internal Medicine, School of Medicine, University of Occupational and Environmental Health, Kitakyushu, Japan; ^2^Mitsubishi Tanabe Pharma, Yokohama, Japan

**Keywords:** systemic lupus erythematosus, toll-like receptor 7, plasmacytoid dendritic cells, interferon-α, toll-like receptor 9

## Abstract

**Objectives:** Aberrant and persistent production of interferon-α (IFN-α) by plasmacytoid dendritic cells (pDCs) is known to play a key role in the pathogenesis of systemic lupus erythematosus (SLE). To assess the precise function of pDCs in SLE patients, we investigated the differential regulation of Toll-like receptor 7 (TLR7) and TLR9 responses during IFN-α production by pDCs.

**Methods:** Peripheral blood mononuclear cells (PBMCs) in SLE patients without hydroxychloroquine treatment, rheumatoid arthritis patients and heathy controls were stimulated with TLR7 and TLR9 agonists. To investigate the priming effect by cytokines, PBMCs from healthy controls were pre-treated with various cytokines and stimulated with TLR7 and TLR9 agonists. The IFN-α production in pDCs was detected by flow cytometry.

**Results:** TLR7-mediated IFN-α production was up-regulated and correlated positively with disease activity in SLE. Conversely, TLR9-mediated IFN-α production was down-regulated. Differential regulation of TLR7/9 response in SLE was independent of TLR7 and TLR9 expression levels. Furthermore, *in vitro* experiments indicated that TLR7-mediated IFN-α production was up-regulated by pre-treatment with type I IFN, whereas TLR9-mediated IFN-α production was down-regulated by pre-treatment with type II IFN.

**Conclusions:** Our study indicates the association between up-regulation of TLR7- mediated IFN-α production by pDCs and disease activity and that TLR7 and TLR9 responses were reversely regulated on pDCs in SLE patients. Thus, type I IFN and TLR7-mediated IFN-α production were involved in a vicious cycle, causing hyper production of IFN-α by pDCs during the pathogenic processes of SLE.

## Introduction

Systemic lupus erythematosus (SLE) is a chronic autoimmune disease with multiple clinical manifestations that differ from one patient to another (1). Although the cause of SLE remains largely unknown, various factors, including genetic and environmental factors, seem to contribute to the pathogenesis of SLE (2, 3). Thus, SLE is an extremely heterogeneous disease in all aspects, but recent studies have demonstrated characteristic induction of type I interferon-regulated genes (IFN-signature), which is linked to a more severe disease activity with organ failure, in patients with SLE (4–6).

Type I IFN, such as IFN-α, is a pleiotropic immunological mediator that bridges the innate and adaptive immunity. Upon viral infection, type I IFN is mainly produced by plasmacytoid dendritic cells (pDCs) when stimulated through Toll-like receptor 7 (TLR7) and TLR9. In SLE patients, abnormal stimulation of TLR7 and TLR9 by self-nucleic acids seems to contribute persistent production of type I IFN. Type I IFN induces aberrant autoantibody production by stimulation of B cells to differentiate into antibody-producing cells and immunoglobulin isotype class-switch and maturation of antigen presenting cells. Thus, type I IFN production by pDCs upon TLR7/9 stimulation has been implicated as a key player in the pathogenesis of SLE. Indeed, targeting type I IFNs and TLR7/9 has recently become a major treatment strategy in SLE (7–10).

To our knowledge, there is a little information on the function of the pDCs in SLE patients. It is reported that the frequency of circulating pDCs is decreased in SLE patients, because activated pDCs seems to infiltrate to inflamed tissue (11–13). On the other hand, functional analysis of circulating pDCs in SLE demonstrated that dysfunctional IFN-α production upon TLR9 stimulation (14). However, the respective impacts of TLR7 and TLR9 response on IFN-α production in SLE have not been addressed. In particular, attention might be paid to drug development by the analysis of TLR7/9 responses, because hydroxychloroquine (HCQ), a known TLR7/9 inhibitor (15), is a mainstay in the current treatment of SLE. Indeed, IFN-α production upon TLR7/9 stimulation is impaired in pDCs from SLE patients who have been treated with HCQ (16).

The main theme of the present study was investigation of the precise function of pDCs in SLE patients without HCQ treatment. Specifically, we determined the differential regulation of TLR7/9 responses during type I IFN production by pDCs. For this purpose, we assessed the TLR7- and TLR9-mediated IFN-α production by pDCs in SLE patients and compared the finding with those in rheumatoid arthritis (RA) patients and healthy controls. In addition, we analyzed the mechanisms of the differential regulation of TLR7/9 responses in SLE patients.

## Methods

### Patients

All cases, who were enrolled in this study, were Asians. SLE patients (n = 68) who fulfilled classification criteria for SLE (17, 18) and who had not been treated with HCQ were enrolled in this study. We also recruited 37 RA patients who fulfilled revised classification criteria for RA (19) and who were not on treatment with biological disease modifying anti-rheumatic drugs (DMARDs), since these drugs are known to influence immunological responses [e.g., anti-TNF Abs are known to induce lupus-like symptoms (20)]. Another control group of 24 healthy subjects free of any autoimmune or infectious disease were recruited to the study (Table 1). The clinical activity of SLE was assessed by the Systemic Lupus Erythematosus Disease Activity Index (SLEDAI) and the British Isles Lupus Assessment Group (BILAG) activity index. Patients with active SLE (aSLE) represented those with more than 10 points on the SLEDAI score, or classified as A1 or B2 by the BILAG index. All other patients who were not labeled as aSLE were grouped into the inactive SLE group (iSLE). The Human Ethics Review Committee of our university reviewed and approved this study, including the collection of peripheral blood samples from the healthy donors and patients. Each subject provided a signed consent form.

**Table 1:** Demographic and clinical characteristics of the study groups.

	**SLE (*n* = 68)**	**RA (*n* = 37)**	**Healthy control (*n* = 24)**
Age (years)	42.0 (15–80)	61.7 (36–80)	35.8 (26–57)
Females, n (%)	61 (90%)	32 (86%)	23 (96%)
Disease duration, years	11.6 (0–30)	5.7 (0–21)	
SLEDAI score	10.1 (0–35)		
SDAI score		28.1 (6.3–99.5)	
BILAG score, A1 or B2, n (%)	42 (62%)		
Anti-dsDNA antibody (U/mL)	85.6 (0–400)		
**Manifestations, n (%)**			
Renal	31 (46%)		
Cutaneous	27 (40%)		
**Treatment, n (%)**			
Hydroxychloroquine	0 (0%)		
Immunosuppressants	36 (53%)		
Corticosteroids	48 (71%)	7 (19%)	
Biological DMARDs		0 (0%)	
Methotrexate		26 (70%)	

Data are number of patients (range) or (percentage).

*SLEDAI score, Systemic Lupus Erythematosus Disease Activity Index; SDAI score, Simple Disease Activity Index; DMARDs, disease modifying antirheumatic drugs*.

### Cell preparation

Peripheral blood mononuclear cells (PBMCs) isolated using Lympholyte-H (Cedarlane) were cultured in complete medium consisting of RPMI1640 supplemented with 10% fetal calf serum, 100 U/mL penicillin, and 100 U/mL streptomycin. Primary human pDCs were purified from PBMCs using Diamond Plasmacytoid Dendritic Cell Isolation Kit II (Miltenyi Biotec), and purity was always >90%. PDCs were cultured in complete medium containing 0.1 ng/mL of recombinant IL-3 (R&D systems).

### TLR stimulation

PBMCs (1 × 10^6^/well) from patients with SLE or RA, or healthy subjects in 48-well plates or pDCs (2.5 × 10^4^/well) from healthy subjects in 96-well flat-bottom plates were stimulated with TLR7 agonist, loxoribine (1 mmol/L) or R837 (5 μg/mL), or TLR9 agonist, CpG2216 (2 μmol/L) or CpG2006 (2 μmol/L, all from InvivoGen) for 5 h, and brefeldin A (2.5 μg/mL: SIGMA-Aldrich) was added during the final 3 h of stimulation to block cytokine secretion. In the case of pre-treatment experiments, PBMCs or pDCs were treated with IFN-α (IFN-α1: Abcam), IFN-β (Peprotech), IFN-γ (R&D systems), TNF-α (Peprotech), IL-6 (Miltenyi Biotech)/soluble IL-6R (R&D systems) or IL-10 (R&D systems) for 2, 12, and 24 h. Cells were washed three times by complete medium to remove cytokines, thereafter, stimulated with TLR agonist as mentioned above (Supplementary Figure S1). Cell number and viability of PBMCs after pre-treatment with each cytokine were calculated under microscope using trypan blue dye.

### Flow cytometry

**Figure 1:**
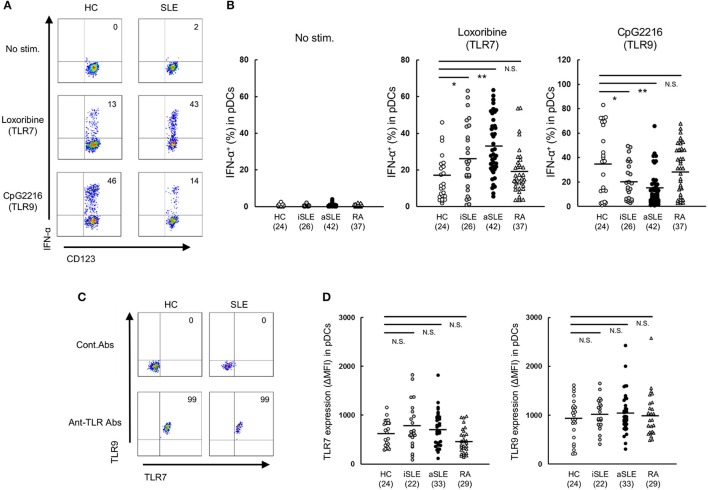
Up-regulation of TLR7-mediated IFN-α production by pDCs in SLE patients. **(A)** Representative flow cytometry plots showing TLR7/9-mediated IFN-α production in Lin^−^ HLA-DR^+^ CD11c^−^ CD123^+^ pDCs from healthy control subjects and SLE patients. **(B)** TLR7/9-mediated IFN-α production in pDCs of each group. **(C)** Representative flow cytometry plots showing TLR7/9 expression in pDCs of healthy control subjects and SLE patients. **(D)** TLR7/9 expression in pDCs of each group. Horizontal lines represent the mean value of each group. *p < 0.05, **p < 0.01, compared to the control (Mann-Whitney's *U*-test). Numbers in brackets on the abscissa represent the number of subjects of each group.

Cells were stained with FITC-conjugated Lineage cocktail 1 (which includes anti-CD3: clone SK7, anti-CD14: clone MΦP9, anti-CD16: clone 3G8, anti-CD19: clone SJ25C1, anti-CD20: clone L27 and anti-CD56: clone NCAM16.2), V500-conjugated anti-HLA-DR (clone G46-6), PE-Cy7-conjugated anti-CD11c (clone B-ly6), and PerCP-Cy5.5-conjugated anti-CD123 (clone 7G3). After fixation and permeabilization with Fixation/Permeabilization buffer (e-Biosciences), the cells were stained with PE-conjugated anti-IFN-α2b (clone 7N4-1), PE-conjugated anti-IFN-α (clone LT27:295 recognize the majority of the IFN-α subtypes, but not IFN-α2b), and FITC-conjugated anti-IFN-β (clone MMHB-1) for intracellular cytokine or PE-conjugated anti-TLR7 (clone 4G6) and APC-conjugated anti-TLR9 (clone eB72-1665) for intracellular TLR. After intracellular staining, cells were analyzed with FACSVerse (BD Biosciences) and FlowJo software (Tomy Digital Biology). Lin^−^ HLA-DR^+^ CD11c^−^ CD123^+^ cells were gated as pDCs (Supplementary Figure S2; confirmed by BDCA2 expression), and cytokine positivity in pDCs was determined as an indicator of cytokine production by pDCs. TLR expression levels in pDCs were analyzed before TLR stimulation and were defined as ΔMFI (MFI of anti-TLR Ab – MFI of isotype control), since almost all pDCs were TLR7/9 positive in all donors (Figure 1C). All antibodies, except anti-IFN-α (clone LT27:295: Miltenyi Biotec), anti-IFN-β (PBL Assay Science) and anti-TLR7 (ThermoFischer), were purchased from BD biosciences and isotype-matched mouse IgG controls (BD biosciences) were used to evaluate the background.

### ELISA for IFN-α in serum

IFN-α concentration in serum were determined using VeriKine-HS Human Interferon Alpha All Subtype ELISA Kit (PBL Assay Science).

### Confocal microscopy

Primary human pDCs were purified from PBMCs using Diamond Plasmacytoid Dendritic Cell Isolation Kit II (Miltenyi Biotec), and purity was always >90%. PDCs were treated with IFN-α for 2 h. Cells were fixed with 4% paraformaldehyde, permeabilized with 0.2% Triton X-100, and then treated with Image-iT FX Signal Enhancer (ThermoFischer) for preventing nonspecific staining. Cells were stained with rabbit anti-TLR7 (polyclonal: Novus Biologicals), mouse anti-EEA1 (1G11: Abcam), mouse anti-Rab7 (Rab7-117: Abcam), rat anti-LAMP-1 (1D4B: Abcam) and goat anti-BDCA2 (polyclonal: R&D systems) as primary Abs and subsequently stained with AlexaFluor488-conjugated anti-rat IgG, AlexaFluor594-conjugated anti-rabbit IgG and AlexaFluor647-conjugated anti-goat IgG (all from Invitrogen) as secondary Abs. Cells were spun onto a microscope slide using the Shandon Cytospin 4 (ThermoFischer) and mounted with Fluoromount/Plus (Diagnostic Biosystems). All samples were visualized using FM10i confocal laser scanning microscope (Olympus), and images were captured and analyzed using FV10-ASW viewer (Olympus). PDCs were identified as BDCA2 positive cells. Pearson's coefficient was calculated for analysis of the co-localization of TLR7 and endosomal markers (EEA1, Rab7, and LAMP1).

### Statistical analysis

Comparison between the disease groups was performed with the nonparametric Mann-Whitney's *U*-test. Correlation analysis was performed with the Spearman's correlation coefficients. In the pre-treatment *in vitro* experiments, data are expressed as mean ± S.E.M. of 3–4 experiments. Differences between groups were examined by the student's *t*-test. A *P*-value less than 0.05 was considered statistically significant. All statistical analyses were conducted using the IBM SPSS software ver. 22.

## Results

### Patients' characteristics

The characteristics of participating subjects are shown in Table 1. All were Asians, and the mean age of SLE was 42.0 years that was matched with healthy donors (mean 35.8 years) but not with RA patients (mean 61.7 years). Most SLE patients were female (90%) that was matched with healthy donors (96%) and with RA patients (86%). The mean duration of illness was 11.6 years in SLE and was 5.7 years in RA. Mean disease activity was 10.1 for SLEDAI in SLE and 28.1 for SDAI in RA. Mean anti-ds DNA antibody titer in SLE was 85.6 U/ml. The proportion of SLE patient with one or more BILAG category A, or two or more BILAG category B was 62%, although concomitant immunosuppressant medication was given.

### TLR7-mediated IFN-α production is up-regulated in pDCs of SLE patients

To clarify the function of pDCs in SLE patients, we assessed TLR7- and TLR9-mediated IFN-α production in pDCs in PBMC from patients with SLE or RA, or healthy subjects. Spontaneous cytokine production without TLR stimulation was marginal in any groups. TLR7-mediated IFN-α production was significantly up-regulated in both inactive and active SLE, but not in RA patients, compared with healthy subjects. TLR7-mediated IFN-α production in active SLE with glomerulonephritis showed higher tendency than those without glomerulonephritis. There was no significant difference in TLR7-mediated IFN-α production among patients under different treatment (Supplementary Figures S3A-D). On the other hand, TLR9-mediated IFN-α production was significantly down-regulated in both inactive and active SLE, compared to healthy subjects (Figures 1A,B), consistent with previous reports (14). These differential TLR7/9 responses in pDCs were specific to SLE patients; they were not observed in RA patients.

To investigate whether the differential regulation of TLR7/9 response in SLE patients is dependent on the expression levels of TLR7 and TLR9, we analyzed TLR7/9 expression in pDCs by flow cytometry. The expression levels of TLR7 and TLR9 in pDCs were comparable among the four groups (Figures 1C,D). Furthermore, there was no correlation between TLR7 expression level and TLR7-mediated IFN-α production in both SLE patients and healthy subjects. On the other hand, TLR9 expression level correlated with TLR9-mediated IFN-α production in healthy controls, but not in SLE patients (Supplementary Figure S4). Taken together, the results indicate that differential regulation of TLR7/9 response in SLE patients did not seem to be dependent on TLR7 and TLR9 expression levels in pDCs.

**Figure 2:**
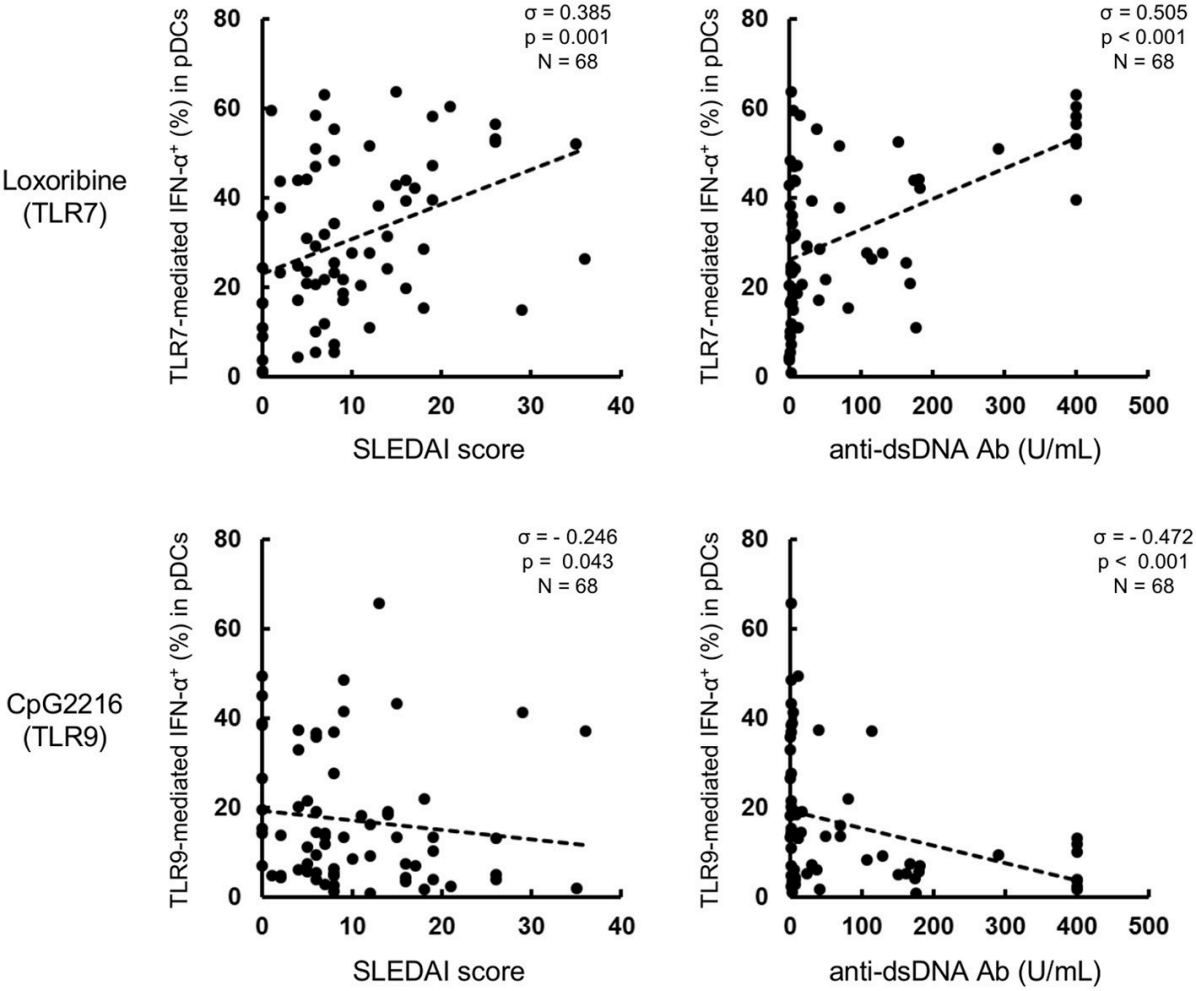
TLR7-mediated IFN-α production correlates with disease activity in SLE patients. Correlation between TLR7/9-mediated IFN-α production and SLEDAI or anti-dsDNA Ab titer. Statistical analysis by the Spearman's correlation coefficient.

Further analysis showed that TLR7-mediated IFN-α production was higher in active than inactive SLE. Furthermore, TLR7-mediated IFN-α production correlated positively with SLEDAI score and anti-dsDNA titers, while TLR9-mediated IFN-α production correlated negatively with disease activity (Figure 2). These results suggest that TLR7-mediated IFN-α production from pDCs is involved in the pathological processes of SLE.

### TLR7-mediated IFN-α production is up-regulated by priming effect of type I IFN, while TLR9-mediated IFN-α production is down-regulated by priming effect of type II IFN

Next, to investigate the mechanisms of the differential regulation of TLR7/9 response in SLE patients, we analyzed the effects of cytokines on TLR7/9 response, since the serum levels of various cytokines (e.g., IFNs) are elevated in SLE patients (21–24). In these experiments (Figure 1), PBMCs were washed away from any soluble factor present in the serum, such as cytokines, during the process of their isolation from whole blood, and thereafter stimulated with TLR7 and TLR9 agonists. To imitate the serum condition in SLE, PBMCs from healthy controls were pre-treated with various cytokines for 24 h, washed to remove cytokines, and thereafter stimulated with TLR7 and TLR9 agonists (Supplementary Figure S1). Interestingly, TLR7-, but not TLR9-, mediated IFN-α production was significantly up-regulated by pre-treatment with type I IFN, such as IFN-α and IFN-β. TLR7-mediated IFN-α production was also up-regulated by pre-treatment with IFN-γ, although the magnitude of up-regulation was less than with type I IFN. Conversely, TLR9-mediated IFN-α production was significantly down-regulated by pre-treatment with IFN-γ. All subtypes of type I IFN production were regulated by pre-treatment with IFN-α, -β, and -γ (Supplementary Figure S5A).

**Figure 3:**
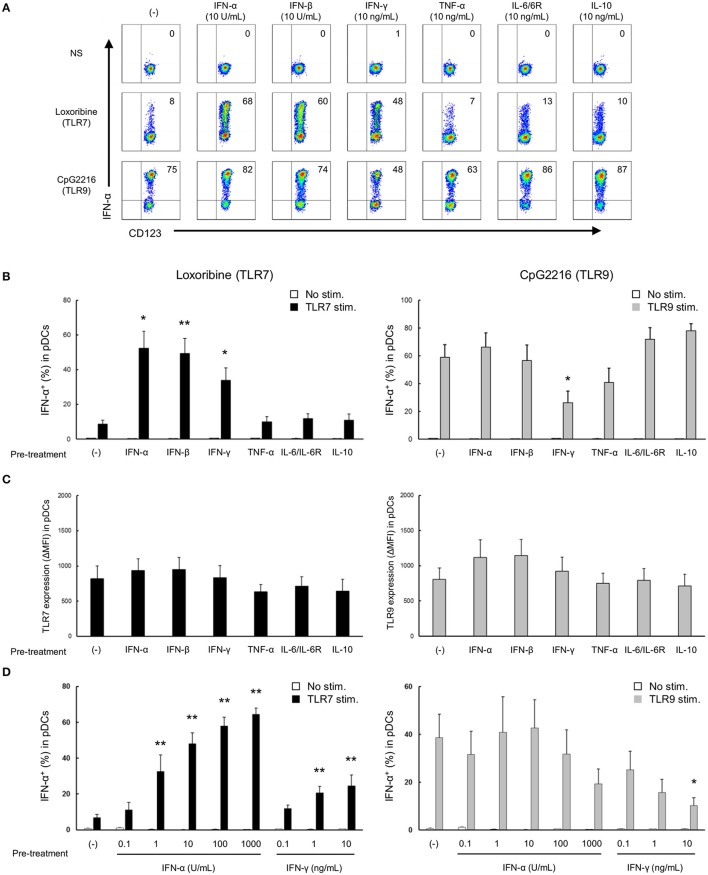
TLR7/9 response of pDCs is regulated by the priming effects of types I and II IFNs in PBMCs of healthy subjects. **(A)** Representative flow cytometry plots showing TLR7/9-mediated IFN-α production in pDCs after pre-treatment with each cytokine. **(B)** TLR7/9-mediated IFN-α production in pDCs after pre-treatment with each cytokine. **(C)** TLR7/9 expression in pDCs after pre-treatment with each cytokine. **(D)** TLR7/9-mediated IFN-α production in pDCs after pre-treatment with different doses of IFN-α and IFN-γ. Data are mean ± S.E.M. of 4 independent experiments. *p < 0.05, **p < 0.01, compared to pre-treatment with media (Student's *t*-test).

TLR7- and TLR9-mediated IFN-α production was not affected by pre-treatment with other cytokines, such as TNF-α, IL-6, and IL-10 (Figures 3A,B). These pre-treatment effects were not due to the effect of survival of pDCs, because no changes were observed in the number and viability of PBMCs, percentage of pDCs in PBMC, and absolute number of pDCs after pre-treatment with any of the above cytokines for 24 h (Supplementary Figure S5B). Furthermore, the expression levels of TLR7 and TLR9 in pDCs were not affected by pre-treatment with any cytokines (Figure 3C). These results demonstrate that TLR7/9 response in pDCs is regulated by the priming effects of both types I and II IFNs without affecting TLR7/9 expression levels.

Moreover, a dose-dependent priming effect was observed for both IFN-α and IFN-γ; notably, TLR7-mediated IFN-α production was significantly up-regulated by pre-treatment with only 1 U/mL of IFN-α. Conversely, IFN-α production following TLR9 stimulation was down-regulated by pre-treatment with higher concentration (10 ng/mL) of IFN-γ (Figure 3D). Furthermore, experiments using R837 and CpG2006, other TLR7 and TLR9 agonists, respectively, confirmed that the specificity of the priming effect of type I IFNs on TLR7 response (Supplementary Figure S6). These results were similar to those on the functional differences in pDCs of SLE patients, suggesting that the differential regulation of TLR7/9 response in pDCs in SLE patients is regulated by both type I and type II IFNs. Indeed, there was a positive correlation between TLR7-mediated IFN-α production and IFN-α concentration in serum, and IFN-α concentration in serum and SLEDAI (Supplementary Figures S7A,B). However, we couldn't show significant correlation between TLR9-mediated IFN-α production and IFN-γ concentration in serum, because IFN-γ in serum were detected in only few patients (data not shown).

**Figure 4:**
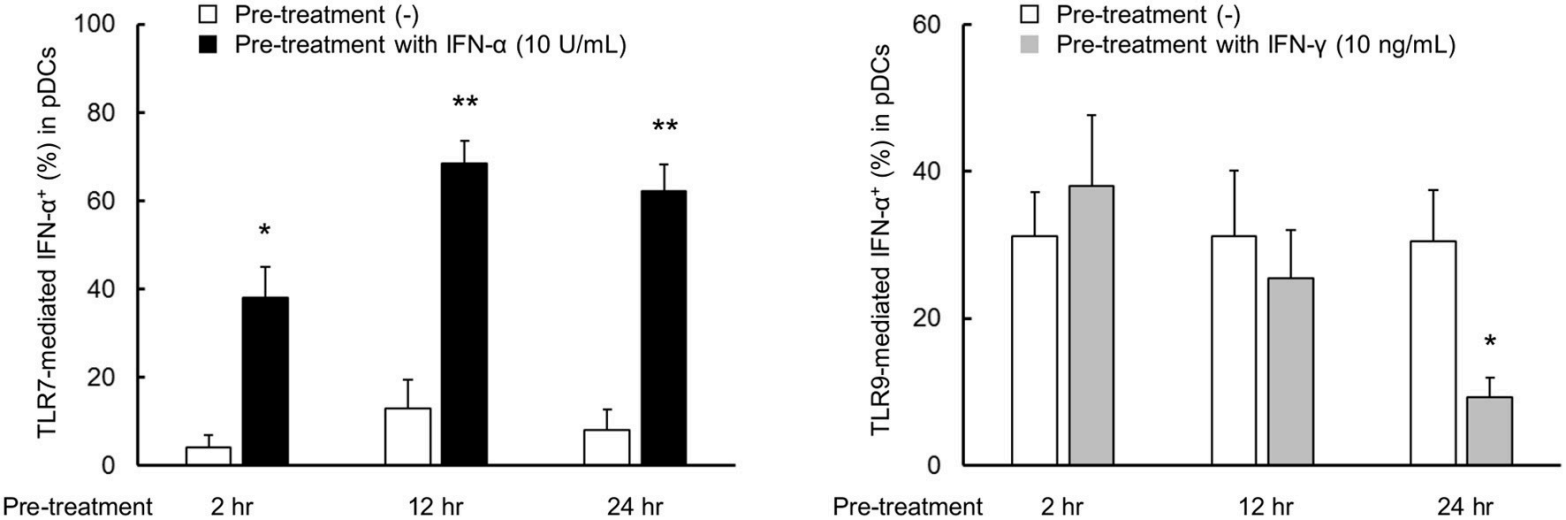
TLR7 response was quickly up-regulated by IFN-α, but down-regulation of TLR9 response by IFN-γ was required long time. PBMCs were pre-treated with IFN-α and IFN-γ for 2, 12, and 24 h, followed by stimulation with TLR7/9 agonist for 5 h. TLR7/9-mediated IFN-α production in pDCs after pre-treatment with each condition. Data are mean ± S.E.M. of 3 independent experiments. *p < 0.05, **p < 0.01, compared to pre-treatment with media (Student's *t*-test).

Next, we performed the time course experiment on pre-treatment effects of IFN-α and IFN-γ on TLR responses. TLR7-mediated IFN-α production was up-regulated after pre-treatment with IFN-α within 2 h (Figure 4). On the other hand, down-regulation of TLR9-mediated IFN-α production by pre-treatment with IFN-γ required for 24 h (Figure 4). Consequently, TLR7 response was quickly up-regulated by IFN-α, but down-regulation of TLR9 response by IFN-γ was required long time.

### Type I and type II IFNs have direct priming effect on purified pDCs

**Figure 5:**
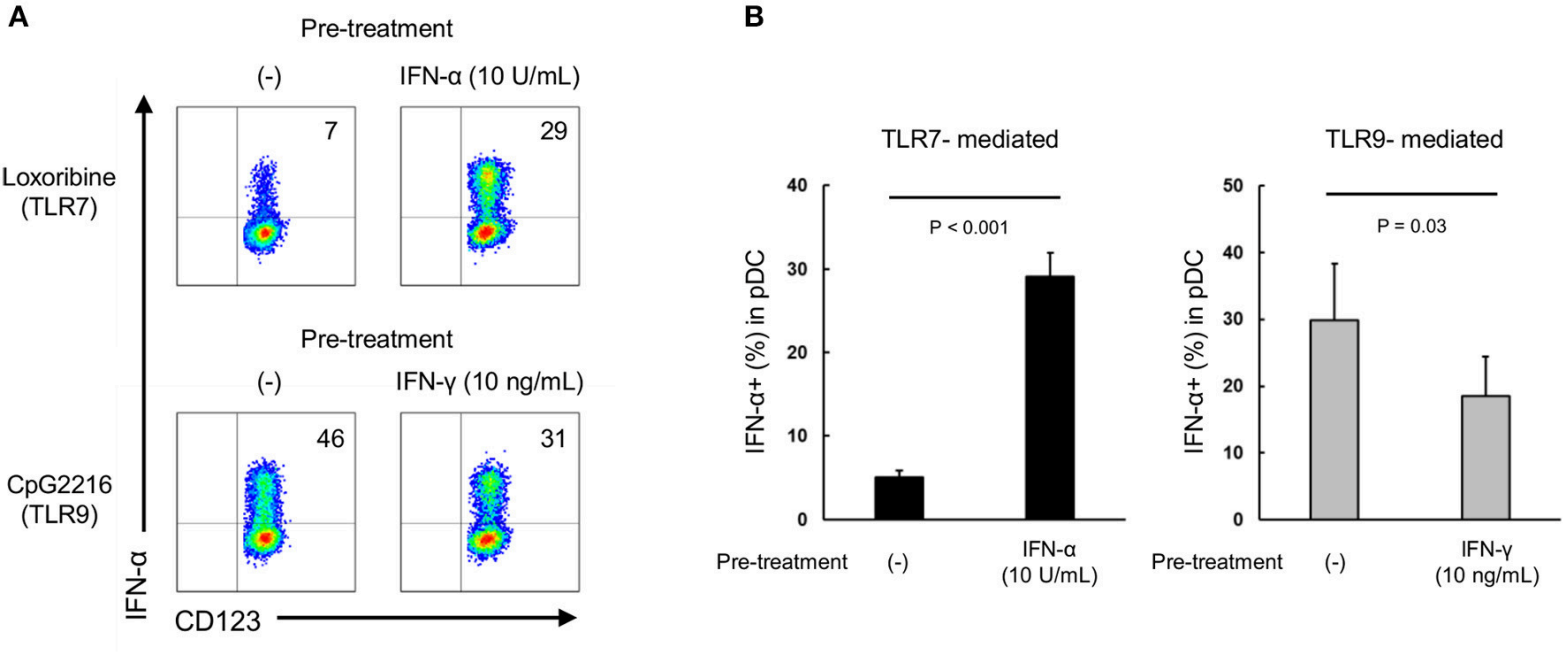
Both IFN-α and IFN-γ have direct priming effect on purified pDCs. **(A)** Representative flow cytometry plots showing TLR7/9-mediated IFN-α production by purified pDCs after pre-treatment with IFN-α and IFN-γ. **(B)** TLR7/9-mediated IFN-α production by purified pDCs after pre-treatment with IFN-α and IFN-γ. Data are mean ± S.E.M. of 3 independent experiments.

We investigated whether type I and type II IFNs have a direct priming effect on purified pDCs. Although IL-3 is generally added in these experiments to maintain survival of cultured purified pDC, it is reported that IL-3 itself up-regulates TLR responses (25). Accordingly, we evaluated the priming effects of IFN-α and IFN-γ on pDCs in the presence of IL-3 (0.1 ng/mL, a concentration that had no effect on TLR7 response (Supplementary Figure S8). TLR7-mediated IFN-α production was up-regulated by pre-treatment with IFN-α, and TLR9-mediated IFN-α production was down-regulated by pre-treatment with IFN-γ (Figures 5A,B). These results indicate that both type I and type II IFNs have direct priming effects on purified pDCs.

### IFN-α increases TLR7 trafficking to lysosome-related organelle

**Figure 6:**
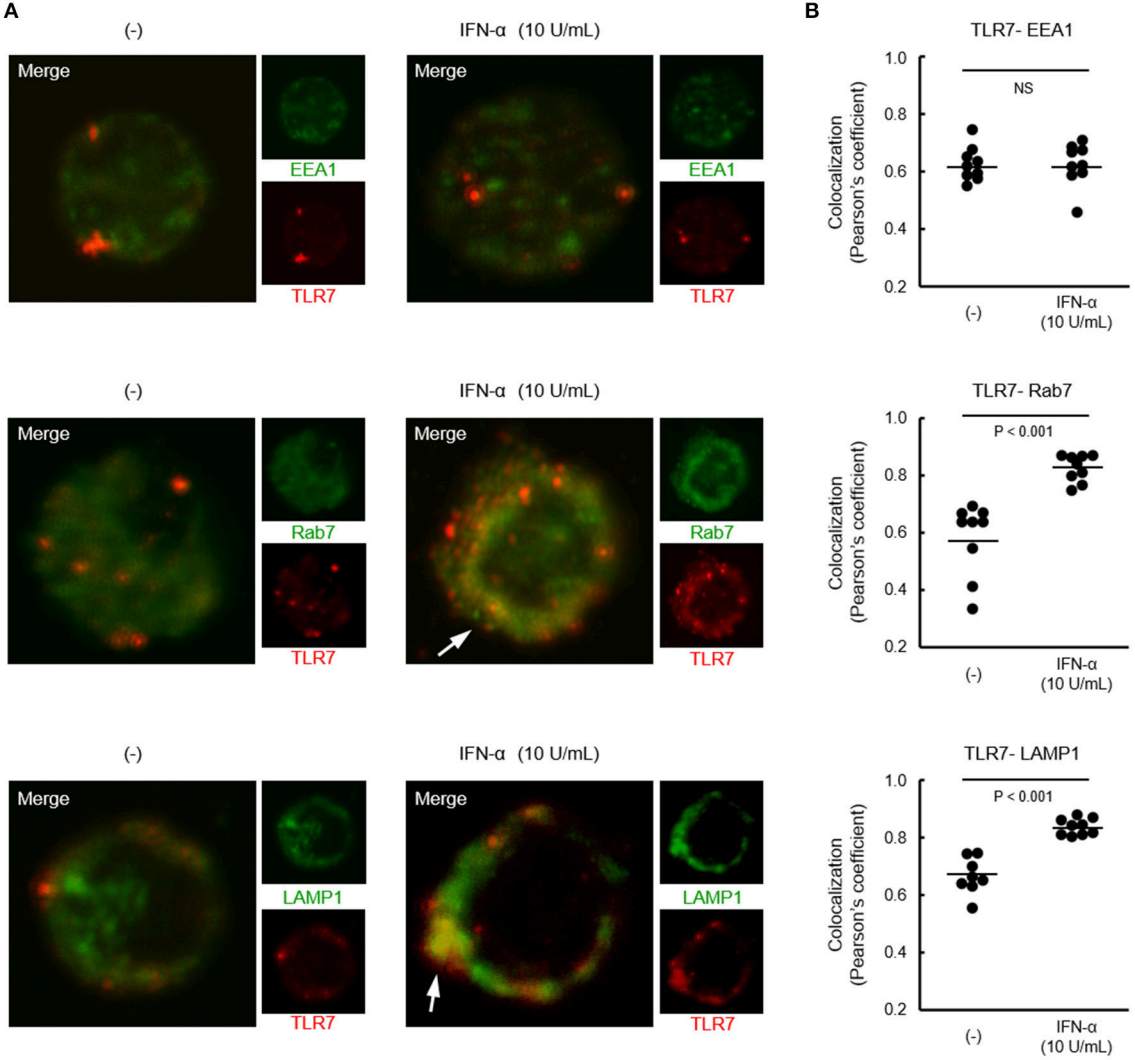
Increased localization of TLR7 in late endosome and lysosome by IFN-α. **(A)** Representative images showing TLR7 (red) and indicated endosomal maturation markers (green) in pDCs pre-treated with or without IFN-α. White arrows indicate robust co-localization of TLR7 with Rab7 and LAMP1. **(B)** Quantification of co-localization between TLR7 and EEA1, Rab7, and LAMP-1. Data shows 10 cells per each condition in one of three independent experiments.

Although both TLR7 and TLR9 located in endosome share signaling molecules, downstream signals were bifurcated dependent on endosomal maturation stage; notably, IFN-α production requires TLR trafficking to lysosome-related organelle (26). Finally, we investigated the localization of TLR7 in pDCs from healthy controls after treatment with IFN-α. Co-localization of TLR7 with Rab7 (late endosome marker) and LAMP1 (lysosome marker), but not with EEA1 (early endosome marker) was increased by pre-treatment with IFN-α (Figure 6). These results demonstrate that increased TLR7 trafficking to lysosome-related organelle by type I IFN may cause the up-regulation of TLR7-mediated IFN-α production in pDCs, without affecting TLR7 expression levels.

## Discussion

The main findings of the present study were that TLR7-mediated IFN-α production was up-regulated in SLE and that the level of production correlated positively with disease activity. Conversely, TLR9-mediated IFN-α production was decreased in SLE patients. Thus, TLR7 and TLR9 responses in pDCs were differentially regulated in SLE. The differential regulation of TLR7/9 response was not dependent on the expression levels of TLR7 and TLR9 in pDCs. The results also showed that such differential regulation of TLR7/9 response in pDCs of SLE patients was due to the priming effects of type I and type II IFNs; namely, TLR7-mediated IFN-α production was up-regulated by pre-treatment with type I IFN and TLR9-mediated IFN-α production was down-regulated by pre-treatment with type II IFN.

Functional studies of purified pDCs from patients with SLE have been hindered by technical limitations, because pDCs are rare cells (<1% in PBMC), and especially, circulating pDCs are reduced in SLE patients (11). Our preliminarily experiments showed that at least 100 mL blood is required to obtain stably, enough and highly purified pDCs from each patient with SLE. It is ethically difficult to collect huge amount of blood from each SLE patient. To overcome these issues, we determined IFN-α positivity in pDCs in PBMC by flow cytometry after stimulation with TLR agonists as an indicator of IFN-α production. By using this method, we found decreased TLR9-mediated IFN-α production from pDCs in SLE patients, consistent with previous report (14). By contrast, we found that TLR7-mediated IFN-α production was significantly up-regulated in SLE patients. Our results also showed that TLR7/9 expression levels in pDCs were similar in SLE patients and healthy controls. Recent studies reported high TLR7/9 expression levels in PBMCs and B cells of SLE patients (22, 27), but the relationship between TLR7/9 expression level and TLR7/9 response remains poorly understood. Zorro et al. (28) demonstrated that increased TLR9 expression had no influence on TLR9 response in B cells from SLE. Our results showed the correlation of TLR9 expression and TLR9 response was observed in the healthy control, but not in SLE patients which probably due to the priming effect of type II IFN. On the other hand, TRL7 expression does not correlate with TLR7 response even in healthy control. In this study, although we showed the priming effects of type I and II IFNs as one of the mechanisms for regulating TLR7/9 responses, TLR7 response in healthy control might be regulated by other mechanisms.

TLR7/9 responses in pDCs are regulated in the presence of certain cytokines, such as IFN-α/β and TNF-α (29–32). Our results showed that both types I and II IFN have priming effects on pDCs, and that the TLR7/9 response is regulated by these cytokines without affecting TLR7/9 expression levels, even after the removal of these cytokines. On the other hand, TNF-α and IL-10 had no priming effect on pDCs, although TLR7/9-mediated IFN-α production was inhibited in the presence of these cytokines (30, 32). IFN signature is probably induced by type II IFN in addition to type I IFN, and both types are elevated in sera of SLE patients (21, 24). Interestingly, no differential regulation of TLR7/9 response was observed in RA patients, in whom IFNs play negligible pathogenic role (6). Considered together, our results suggest that differential regulation of TLR7/9 response in pDCs of SLE patients is mediated through the priming effects of types I and II IFNs. We confirmed the positive correlation between TLR7-mediated IFN-α production and IFN-α concentration in serum from patients with SLE.

It is noteworthy that TLR7-mediated IFN-α production was quickly up-regulated after pre-treatment with low concentration of IFN-α (10 U/mL) within 2 h, by the modulation of TLR7 trafficking to lysosome-related organelle, without affecting TLR7 expression levels. More recently, it is reported that increased TLR7 co-localization with Rab7 and LAMP1 in pDCs from patients with SLE (33), probably due to the priming effect of type I IFN. Conversely, down-regulation of TLR9 response was required long time (approximately 24 h) after pre-treatment with high concentration of IFN-γ (10 ng/mL). Although we investigated the effect of IFN-γ on TLR9 trafficking, co-localization of TLR9 with any endosome markers were not influenced by treatment with IFN-γ (data not shown). TLR signaling is regulated by multilayer control mechanisms, including cellular trafficking, cooperation with coreceptors, cleavage and interaction of signaling molecules with negative regulators (34). Thus, further analysis is required for unveiling the precise mechanisms of the differential regulation of TLR7/9 responses in patients with SLE.

Finally, (1) TLR7-mediated IFN-α production was positively correlated with SLE disease activity, (2) TLR7-mediated IFN-α production was positively correlated with IFN-α concentration in serum and (3) IFN-α concentration in serum was positively correlated with SLE disease activity. Furthermore, TLR7-mediated IFN-α production were up-regulated by type I IFN itself. Taken together, these results suggest that TLR7-mediated IFN-α production from pDCs play a pivotal role in the pathogenesis of SLE. In contrast, TLR9-mediated IFN-α production was negatively correlated with SLE disease activity. While the pathological role of TLR7 in human SLE and lupus nephritis in murine models is relatively accepted, the role of TLR9 remains controversial. Several murine studies have highlighted the importance of TLR7, and that TLR9 surprisingly provides protection, in lupus pathogenesis (35, 36). In this study, TLR9-mediated IFN-α production was down-regulated, but still detected in SLE patients. Although the precise role of TLR9 in SLE remains unclear, TLR7 might be more important than TLR9 in the pathogenesis of SLE. In this study, synthetic TLR agonists were used instead of physiological immune complex such as dsDNA-IC or RNP-IC. Since main trigger of TLR7/9 responses in SLE is immune complex with self-nucleic acids, we had attempted to assess the pDC response by RNA-IC stimulation. Unfortunately, we could not detect IFN-α positive pDCs after 5 h stimulation with RNA-IC. It may be due to limitation of the culture period, because more longer culture caused breakdown of gating strategy, especially pDC marker such as CD123 or BDCA2 were down regulated. Consequently, we could not technically distinguish pDCs among PBMCs after longer culture. In addition, our study was limited on SLE in Asia, particularly in Japanese, which may be more interferonopathy than other races. Further investigation will be necessary for a better understanding of the function of pDCs in SLE patients.

In summary, our results suggest that type I IFN and TLR7-mediated IFN-α production establish a vicious cycle, causing aberrant and persistent production of type I IFN in the pathogenic process of SLE. Although SLE is a complex autoimmune disease with extremely heterogeneous clinical manifestations as well as pathogenesis, TLR7 seems to play a pivotal role in the pathogenesis of SLE. Furthermore, TLR7 and TLR9 responses were reversely or differentially regulated on pDCs in SLE, implying that for the pharmaceutical application, TLR7, but not TLR9, should be targeted when targeted therapies are developed in patients with SLE.

## Author contributions

KS and SN designed the research. KS conducted experiments, analyzed data and wrote the manuscript. YM, SK, AI and KN helped to conduct experiments. YT created the research concept and supervised the research and the manuscript.

### Conflict of interest statement

KS and AI are employees of Mitsubishi Tanabe Pharma. YT received grants and research support from Mitsubishi-Tanabe, Takeda, Daiichi-Sankyo, Chugai, Bristol-Myers, MSD, Astellas, Abbvie, Eisai. The remaining authors declare that the research was conducted in the absence of any commercial or financial relationships that could be construed as a potential conflict of interest.

The reviewer KY declared a past co-authorship with one of the authors YT.

## Supplementary material

Supplementary Figure S1 Procedures for evaluating TLR7/9 responses in patients samples and *in vitro* pre-treatment experiment. HC, healthy control subjects.

Supplementary Figure S2 Gating strategy for pDC from PBMC.

Supplementary Figure S3 TLR7/9-mediated IFN-α production in pDCs of each group with or without lupus nephritis **(A)** and with receiving different medications among whole SLE **(B)**, active SLE **(C)**, and active SLE with lupus nephritis **(D)**. Horizontal lines represent the mean value of each group. ^*^*p* < 0.05, ^**^*p* < 0.01, compared to the control (Mann-Whitney's *U*-test).

Supplementary Figure S4 Relationship between TLR7/9-mediated IFN-α production and TLR7 or TLR9 expression levels in pDCs of healthy control subjects (HC) and SLE patients. Statistical analysis with the Spearman's correlation coefficient.

Supplementary Figure S5 Pre-treatment effects of types I and II IFNs on TLR7/9 responses in pDCs. **(A)** All subtypes of type I IFN production were regulated by pre-treatment with IFN-α, -β, and -γ. **(B)** The effects of pre-treatment of types I and II IFNs are not due to the effect of survival of pDCs. Number and viability of PBMCs, percentage of pDCs among PBMC and absolute number of pDCs after pre-treatment with each cytokine for 24 h. ^*^*p* < 0.05, ^**^*p* < 0.01, compared to pre-treatment with media (Student's *t*-test).

Supplementary Figure S6 TLR7 specificity in the priming effect of type I IFN. The percentages of IFN-α producing pDCs stimulated with R837 and CpG2006 after pre-treatment with each cytokine for 24 h. ^*^*p* < 0.05, ^**^*p* < 0.01, compared to pre-treatment with media (Student's *t*-test).

Supplementary Figure S7 Relationship between TLR7-mediated IFN-α production **(A)** and IFN-α levels in serum **(B)** of SLE patients. Statistical analysis with the Spearman's correlation coefficient.

Supplementary Figure S8 TLR7/9-mediated IFN-α production were not affected by IL-3 at the concentration of 0.1 ng/mL. Percentages of IFN-α-producing pDCs stimulated with TLR7 agonist, loxoribine, and TLR9 agonist, CpG2216, for 5 h before and after pre-treatment with IL-3 (0.1 ng/mL) for 24 h. ^*^*p* < 0.05, ^**^*p* < 0.01, compared to pre-treatment with media (Student's *t*-test).
