# Supplementary material for: Up-Regulation of TLR7-Mediated IFN-α Production by Plasmacytoid Dendritic Cells in Patients With Systemic Lupus Erythematosus
Source: Front Immunol. 2018 Aug 28;9:1957. doi: 10.3389/fimmu.2018.01957 (PMC6121190; doi:10.3389/fimmu.2018.01957)
Supplement: Supplementary Figure S1 — Procedures for evaluating TLR7/9 responses in patients samples and in vitro pre-treatment experiment. HC, healthy control subjects. [file Presentation_1.PPTX]

## Slide 1
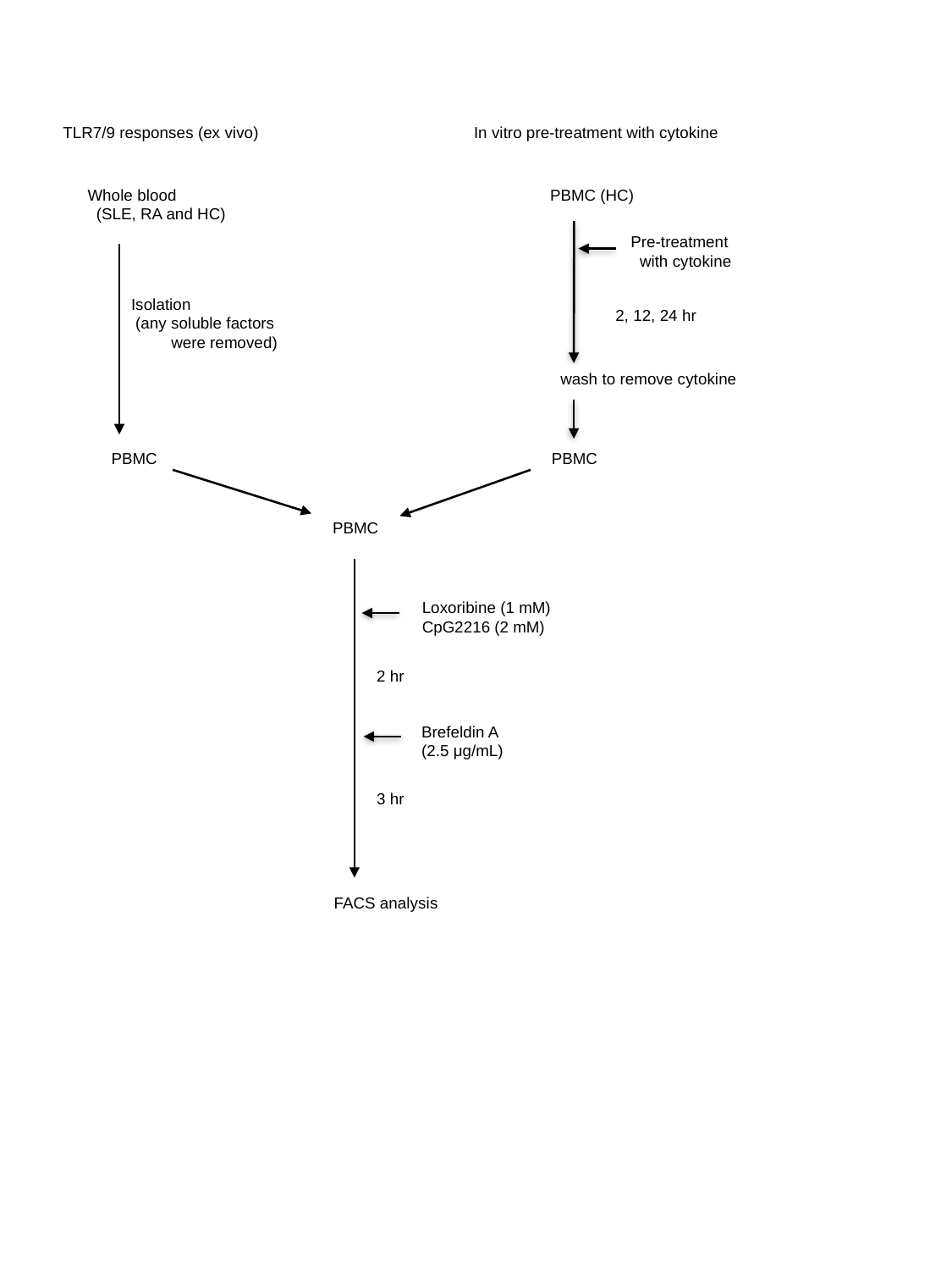

TLR7/9 responses (ex vivo)
In vitro pre-treatment with cytokine
Whole blood
 (SLE, RA and HC)
PBMC (HC)
Pre-treatment
 with cytokine
Isolation
 (any soluble factors
 were removed)
2, 12, 24 hr
wash to remove cytokine
PBMC
PBMC
PBMC
Loxoribine (1 mM)
CpG2216 (2 mM)
2 hr
Brefeldin A
(2.5 μg/mL)
3 hr
FACS analysis
