# Supplementary figures and images for: Up-Regulation of TLR7-Mediated IFN-α Production by Plasmacytoid Dendritic Cells in Patients With Systemic Lupus Erythematosus
Source: Front Immunol. 2018 Aug 28;9:1957. doi: 10.3389/fimmu.2018.01957 (PMC6121190; doi:10.3389/fimmu.2018.01957)

## Slide 1
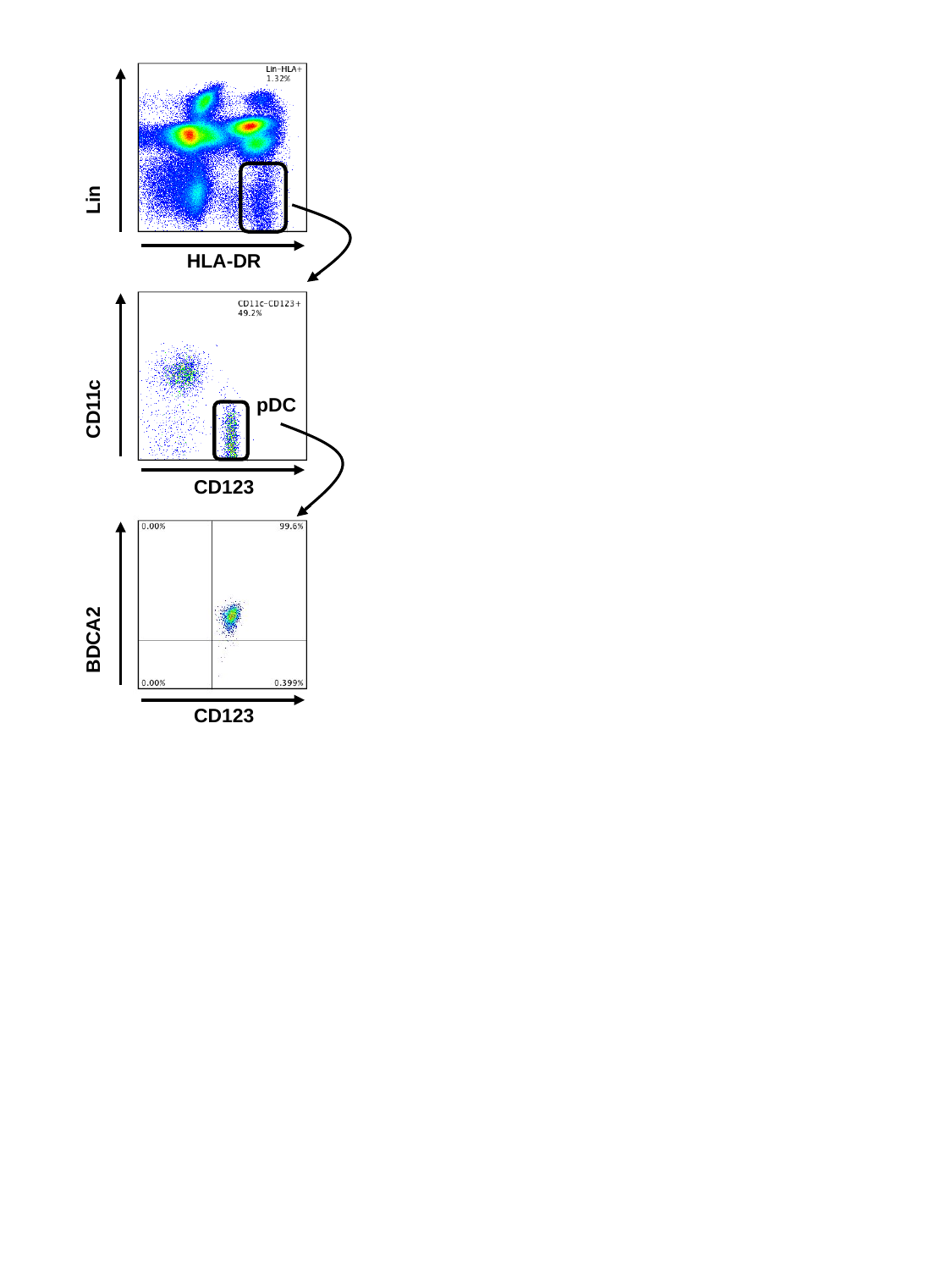

Lin
HLA-DR
CD11c
pDC
CD123
BDCA2
CD123

Supplement: Supplementary Figure S2 — Gating strategy for pDC from PBMC. [file Presentation_2.PPTX]

## Slide 1
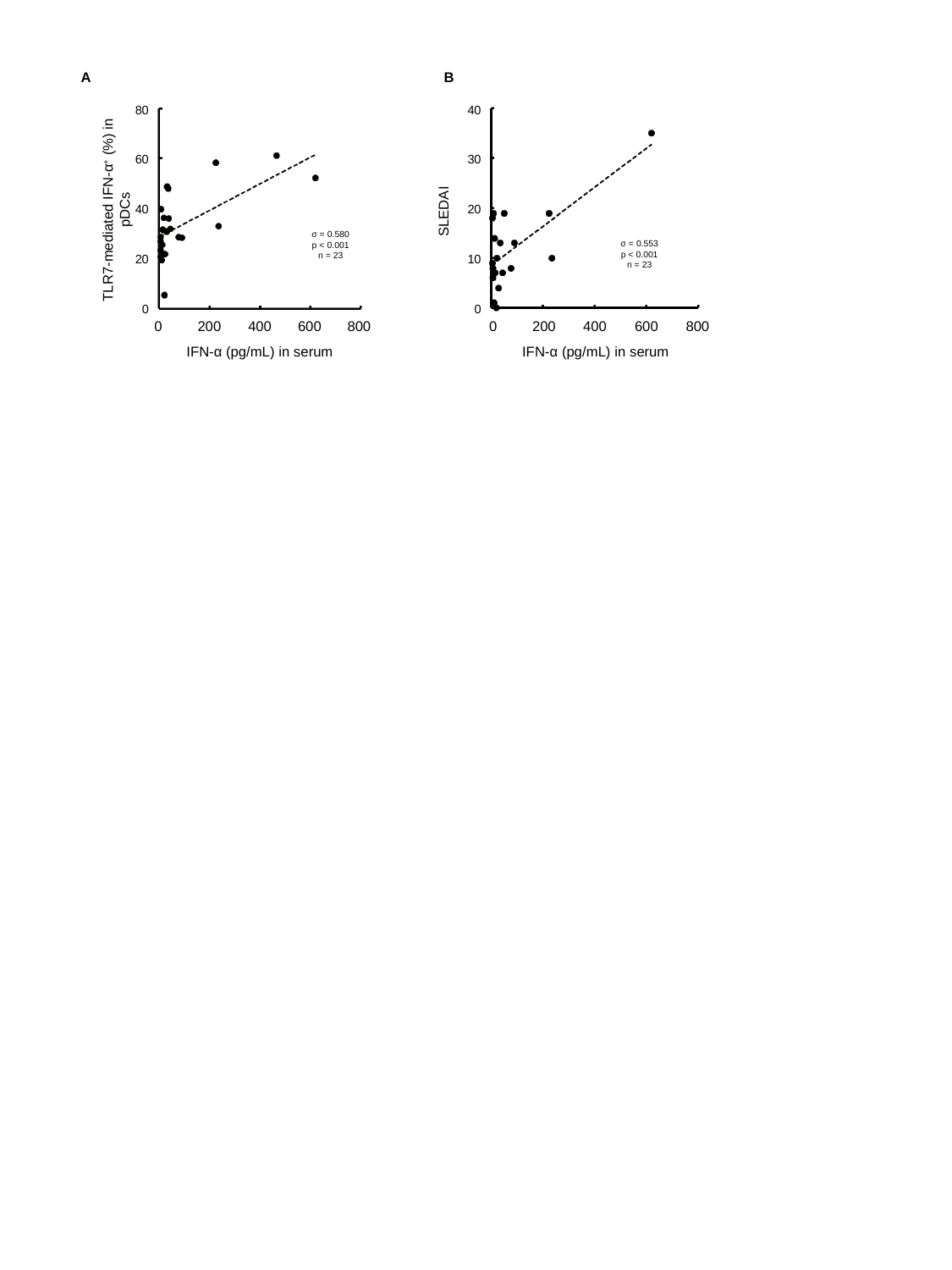

A
B
TLR7-mediated IFN-α+ (%) in pDCs
80
60
40
20
0
40
30
SLEDAI
20
σ = 0.580
p < 0.001
n = 23
σ = 0.553
p < 0.001
n = 23
10
0
0
200
400
600
800
0
200
400
600
800
IFN-α (pg/mL) in serum
IFN-α (pg/mL) in serum

Supplement: Supplementary Figure S7 — Relationship between TLR7-mediated IFN-α production (A) and IFN-α levels in serum (B) of SLE patients. Statistical analysis with the Spearman's correlation coefficient. [file Presentation_7.PPTX]
