# Supplementary material for: Up-Regulation of TLR7-Mediated IFN-α Production by Plasmacytoid Dendritic Cells in Patients With Systemic Lupus Erythematosus
Source: Front Immunol. 2018 Aug 28;9:1957. doi: 10.3389/fimmu.2018.01957 (PMC6121190; doi:10.3389/fimmu.2018.01957)
Supplement: Supplementary Figure S3 — TLR7/9-mediated IFN-α production in pDCs of each group with or without lupus nephritis (A) and with receiving different medications among whole SLE (B), active SLE (C), and active SLE with lupus nephritis (D). Horizontal lines represent the mean value of each group. *p < 0.05, **p < 0.01, compared to the control (Mann-Whitney's U-test). [file Presentation_3.PPTX]

## Slide 1
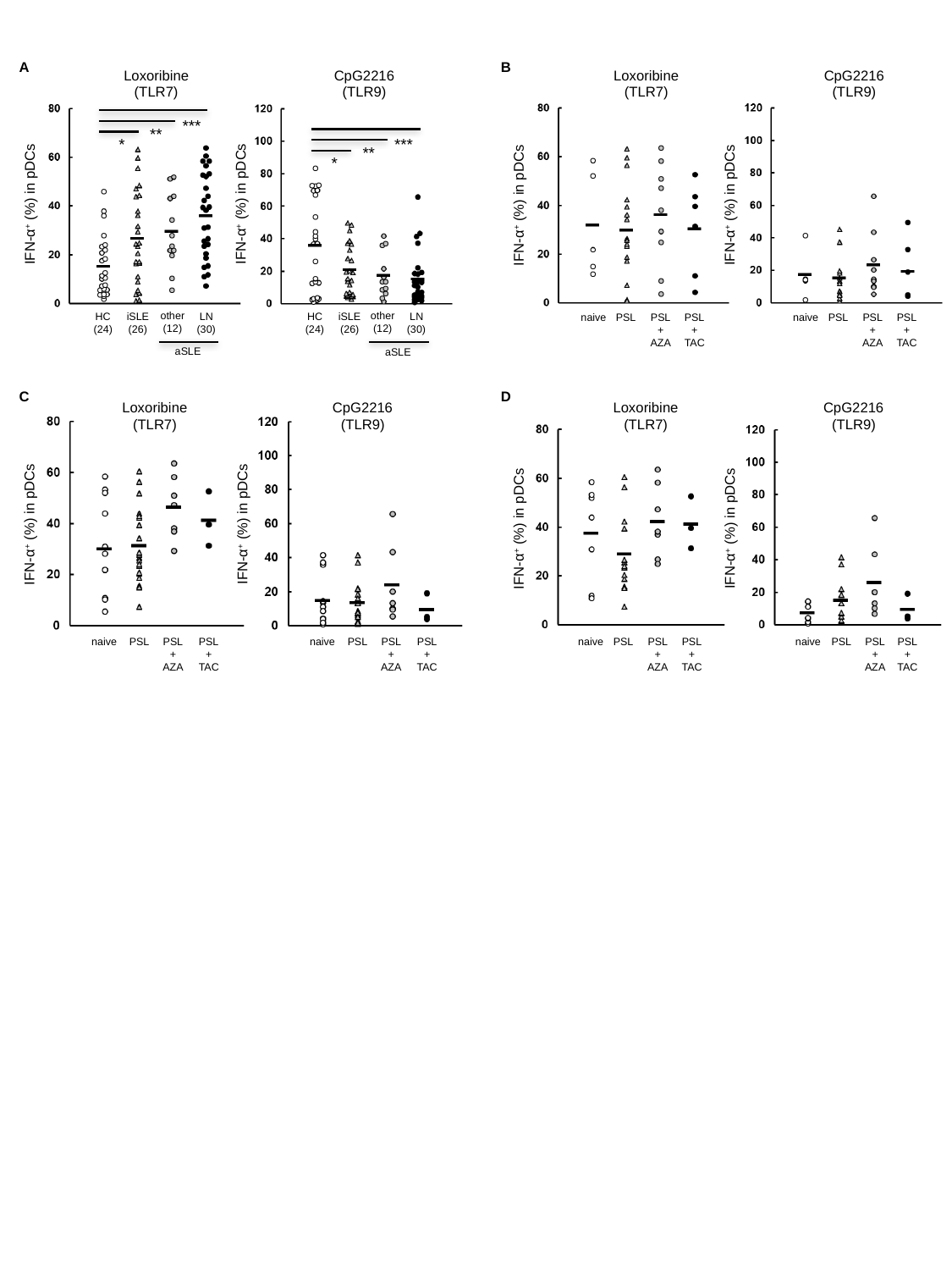

A
B
Loxoribine
(TLR7)
CpG2216
(TLR9)
Loxoribine
(TLR7)
CpG2216
(TLR9)
***
**
IFN-α+ (%) in pDCs
IFN-α+ (%) in pDCs
*
***
IFN-α+ (%) in pDCs
IFN-α+ (%) in pDCs
**
*
other
(12)
other
(12)
HC
(24)
iSLE
(26)
LN
(30)
HC
(24)
iSLE
(26)
LN
(30)
naive
PSL
PSL
+
AZA
PSL
+
TAC
naive
PSL
PSL
+
AZA
PSL
+
TAC
aSLE
aSLE
C
D
Loxoribine
(TLR7)
CpG2216
(TLR9)
Loxoribine
(TLR7)
CpG2216
(TLR9)
IFN-α+ (%) in pDCs
IFN-α+ (%) in pDCs
IFN-α+ (%) in pDCs
IFN-α+ (%) in pDCs
naive
PSL
PSL
+
AZA
PSL
+
TAC
naive
PSL
PSL
+
AZA
PSL
+
TAC
naive
PSL
PSL
+
AZA
PSL
+
TAC
naive
PSL
PSL
+
AZA
PSL
+
TAC
