# Supplementary material for: Up-Regulation of TLR7-Mediated IFN-α Production by Plasmacytoid Dendritic Cells in Patients With Systemic Lupus Erythematosus
Source: Front Immunol. 2018 Aug 28;9:1957. doi: 10.3389/fimmu.2018.01957 (PMC6121190; doi:10.3389/fimmu.2018.01957)
Supplement: Supplementary Figure S4 — Relationship between TLR7/9-mediated IFN-α production and TLR7 or TLR9 expression levels in pDCs of healthy control subjects (HC) and SLE patients. Statistical analysis with the Spearman's correlation coefficient. [file Presentation_4.PPTX]

## Slide 1
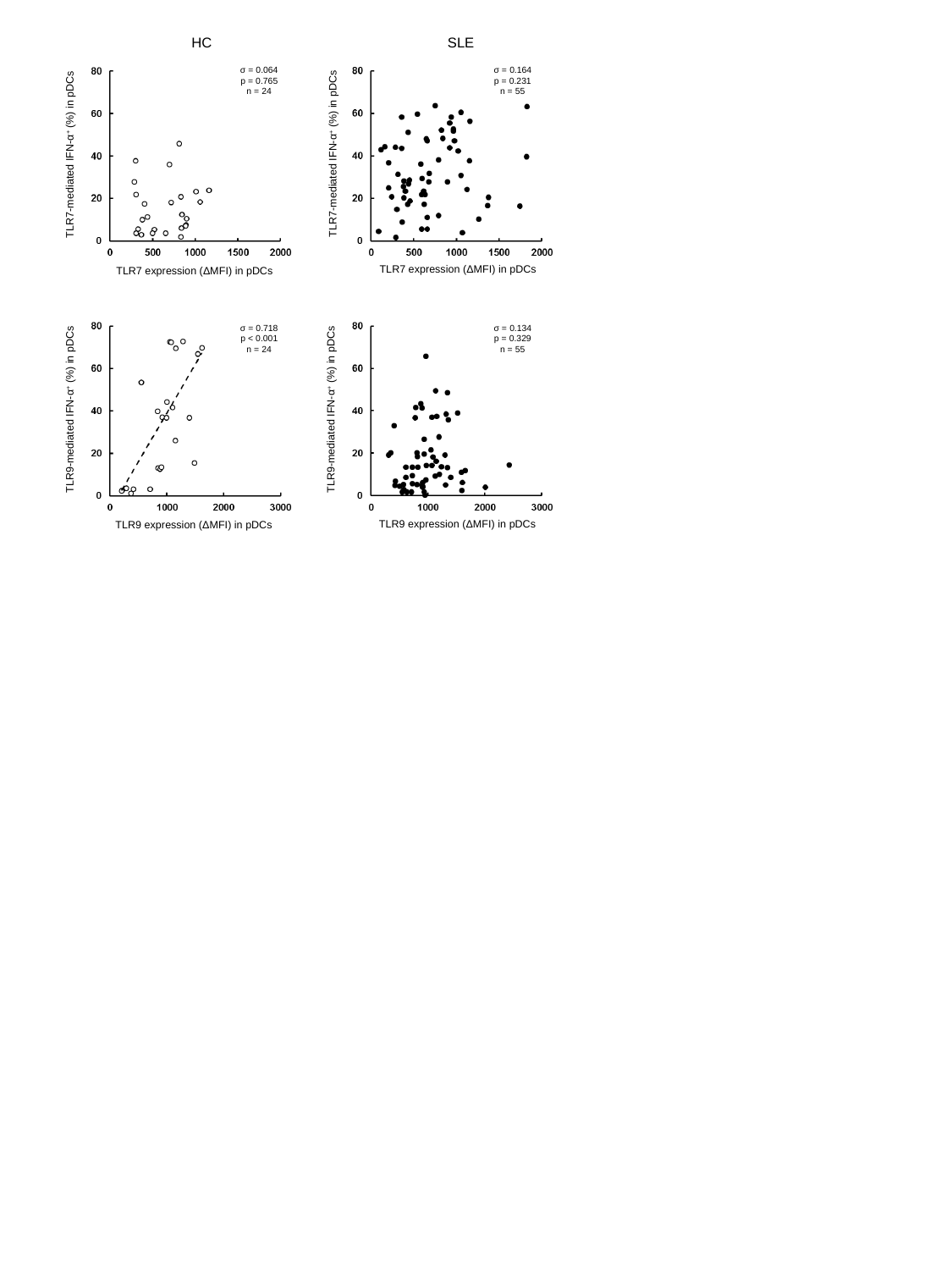

HC
SLE
TLR7-mediated IFN-α+ (%) in pDCs
TLR7-mediated IFN-α+ (%) in pDCs
σ = 0.064
p = 0.765
n = 24
σ = 0.164
p = 0.231
n = 55
TLR7 expression (ΔMFI) in pDCs
TLR7 expression (ΔMFI) in pDCs
TLR9-mediated IFN-α+ (%) in pDCs
TLR9-mediated IFN-α+ (%) in pDCs
σ = 0.718
p < 0.001
n = 24
σ = 0.134
p = 0.329
n = 55
TLR9 expression (ΔMFI) in pDCs
TLR9 expression (ΔMFI) in pDCs
