# Supplementary material for: Up-Regulation of TLR7-Mediated IFN-α Production by Plasmacytoid Dendritic Cells in Patients With Systemic Lupus Erythematosus
Source: Front Immunol. 2018 Aug 28;9:1957. doi: 10.3389/fimmu.2018.01957 (PMC6121190; doi:10.3389/fimmu.2018.01957)
Supplement: Supplementary Figure S5 — Pre-treatment effects of types I and II IFNs on TLR7/9 responses in pDCs. (A) All subtypes of type I IFN production were regulated by pre-treatment with IFN-α, -β, and -γ. (B) The effects of pre-treatment of types I and II IFNs are not due to the effect of survival of pDCs. Number and viability of PBMCs, percentage of pDCs among PBMC and absolute number of pDCs after pre-treatment with each cytokine for 24 h. *p < 0.05, **p < 0.01, compared to pre-treatment with media (Student's t-test). [file Presentation_5.PPTX]

## Slide 1
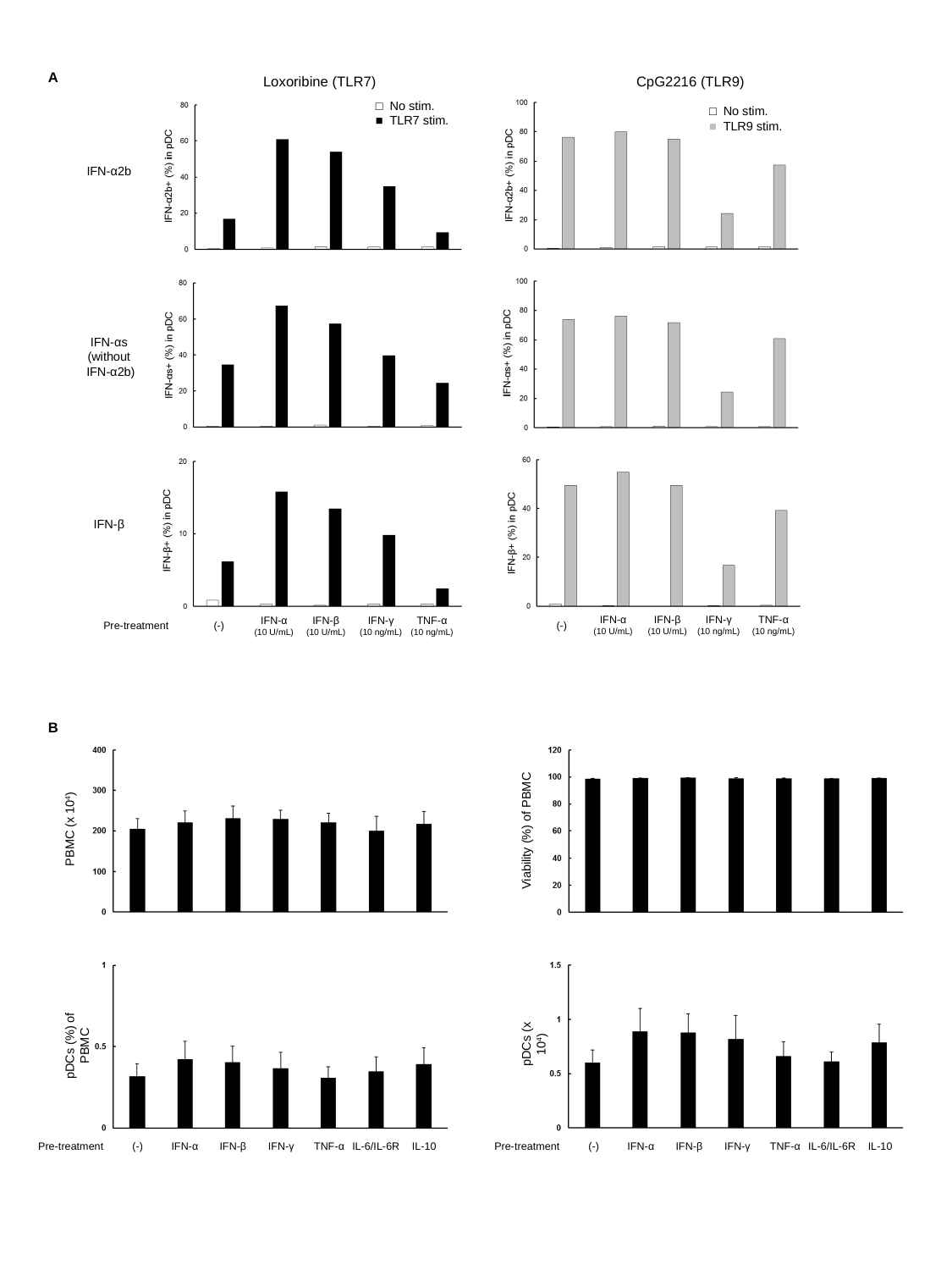

A
Loxoribine (TLR7)
CpG2216 (TLR9)
□ No stim.
■ TLR7 stim.
□ No stim.
■ TLR9 stim.
IFN-α2b
IFN-αs
(without
 IFN-α2b)
IFN-β
IFN-α
(10 U/mL)
IFN-β
(10 U/mL)
IFN-γ
(10 ng/mL)
TNF-α
(10 ng/mL)
IFN-α
(10 U/mL)
IFN-β
(10 U/mL)
IFN-γ
(10 ng/mL)
TNF-α
(10 ng/mL)
(-)
Pre-treatment
(-)
B
Viability (%) of PBMC
PBMC (x 104)
pDCs (%) of PBMC
pDCs (x 104)
Pre-treatment
(-)
IFN-α
IFN-β
IFN-γ
TNF-α
IL-6/IL-6R
IL-10
Pre-treatment
(-)
IFN-α
IFN-β
IFN-γ
TNF-α
IL-6/IL-6R
IL-10
