# Supplementary material for: Up-Regulation of TLR7-Mediated IFN-α Production by Plasmacytoid Dendritic Cells in Patients With Systemic Lupus Erythematosus
Source: Front Immunol. 2018 Aug 28;9:1957. doi: 10.3389/fimmu.2018.01957 (PMC6121190; doi:10.3389/fimmu.2018.01957)
Supplement: Supplementary Figure S6 — TLR7 specificity in the priming effect of type I IFN. The percentages of IFN-α producing pDCs stimulated with R837 and CpG2006 after pre-treatment with each cytokine for 24 h. *p < 0.05, **p < 0.01, compared to pre-treatment with media (Student's t-test). [file Presentation_6.PPTX]

## Slide 1
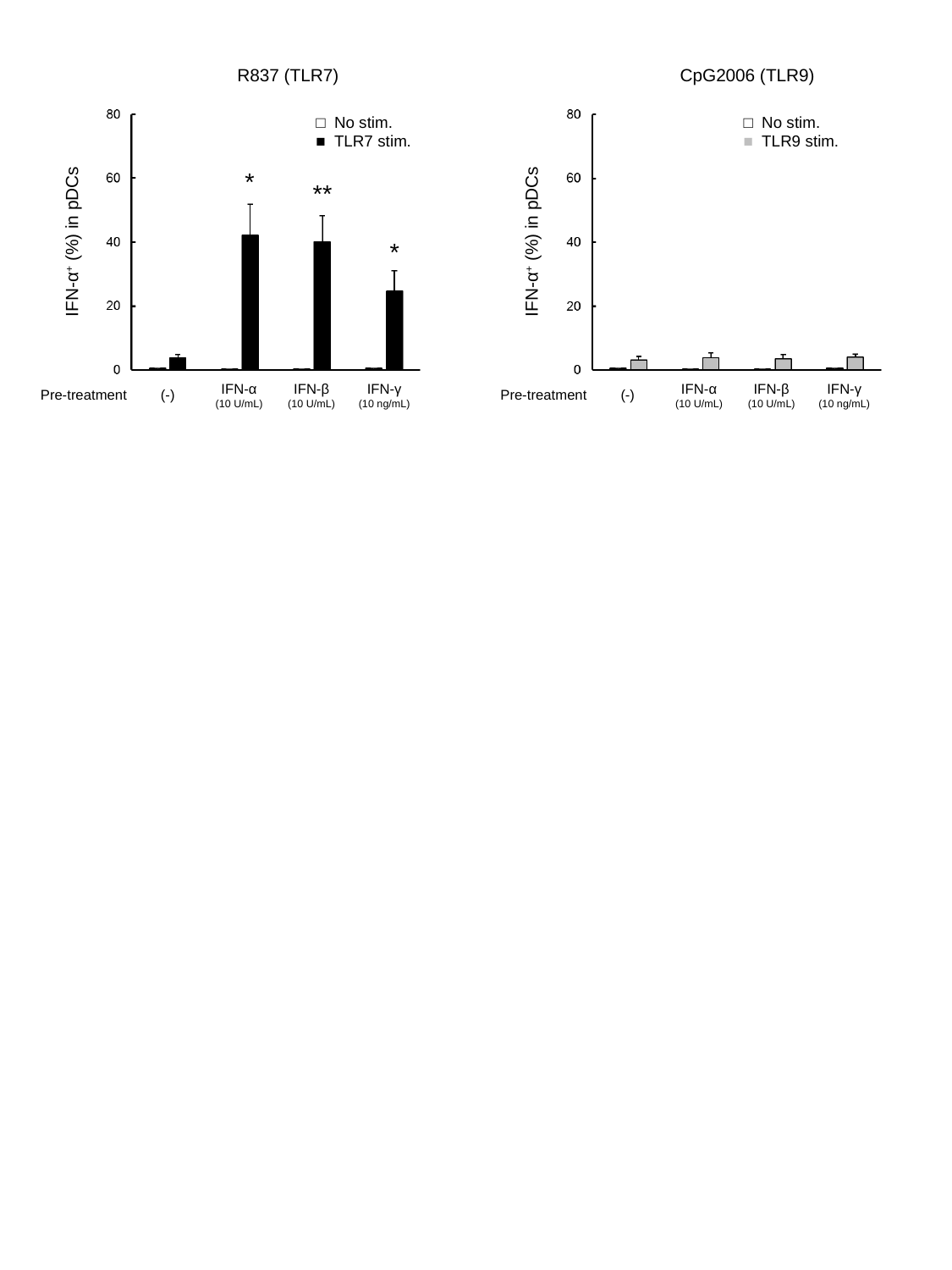

R837 (TLR7)
CpG2006 (TLR9)
□ No stim.
■ TLR7 stim.
□ No stim.
■ TLR9 stim.
IFN-α+ (%) in pDCs
IFN-α+ (%) in pDCs
*
**
*
IFN-α
(10 U/mL)
IFN-β
(10 U/mL)
IFN-γ
(10 ng/mL)
IFN-α
(10 U/mL)
IFN-β
(10 U/mL)
IFN-γ
(10 ng/mL)
Pre-treatment
(-)
Pre-treatment
(-)
