# Supplementary material for: Up-Regulation of TLR7-Mediated IFN-α Production by Plasmacytoid Dendritic Cells in Patients With Systemic Lupus Erythematosus
Source: Front Immunol. 2018 Aug 28;9:1957. doi: 10.3389/fimmu.2018.01957 (PMC6121190; doi:10.3389/fimmu.2018.01957)
Supplement: Supplementary Figure S8 — TLR7/9-mediated IFN-α production were not affected by IL-3 at the concentration of 0.1 ng/mL. Percentages of IFN-α-producing pDCs stimulated with TLR7 agonist, loxoribine, and TLR9 agonist, CpG2216, for 5 h before and after pre-treatment with IL-3 (0.1 ng/mL) for 24 h. *p < 0.05, **p < 0.01, compared to pre-treatment with media (Student's t-test). [file Presentation_8.PPTX]

## Slide 1
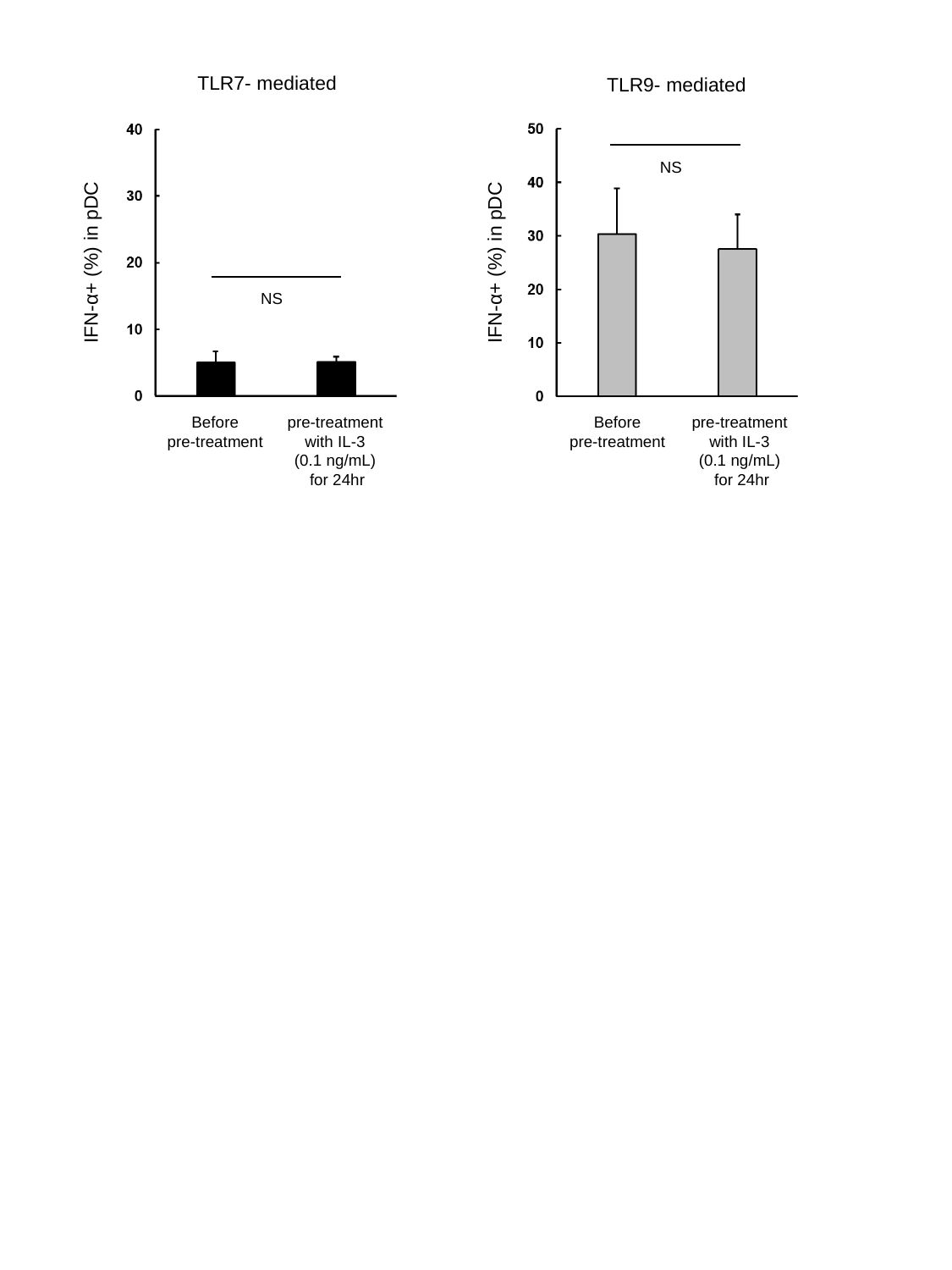

TLR7- mediated
TLR9- mediated
NS
IFN-α+ (%) in pDC
IFN-α+ (%) in pDC
NS
Before
pre-treatment
pre-treatment
with IL-3
(0.1 ng/mL)
 for 24hr
Before
pre-treatment
pre-treatment
with IL-3
(0.1 ng/mL)
 for 24hr
